# Quality indicators for home‐ and community‐based aged care: A critical literature review to inform policy directions

**DOI:** 10.1111/ajag.13103

**Published:** 2022-07-03

**Authors:** Hui Yuan Foong, Joyce Siette, Mikaela Jorgensen

**Affiliations:** ^1^ Centre for Health Systems and Safety Research, Australian Institute of Health Innovation Macquarie University Sydney New South Wales Australia; ^2^ Centre for Ageing, Cognition and Wellbeing Macquarie University Sydney New South Wales Australia

**Keywords:** home care, home‐based aged care, quality indicators, quality measures, social care

## Abstract

**Objectives:**

Australia is lagging behind other countries in implementing quality indicators (QIs) in home‐ and community‐based aged care. This research aimed to identify and appraise home care QI sets used internationally for older adults, to inform the future development and utilisation of QIs in the Australian context.

**Methods:**

A systematic search of eligible studies outlining the development and validation of home care QI sets for older adults was undertaken. QIs were categorised using the Donabedian model to identify potential gaps in coverage of key areas of care quality. Each QI was classified as potentially “derivable” or not from existing national routinely collected datasets. Methodological quality was determined using the Appraisal of Indicators through Research and Evaluation instrument.

**Results:**

Three sets of home care QIs developed and used internationally for older adults were identified. Two of the QI sets focused predominantly on clinical and functional aspects of care. Of 45 unique QIs, the majority were outcome measures (93%), with only three QIs measuring care processes (7%), and zero indicators measuring quality in terms of the structure of care (e.g., waiting time to access services). Nearly half of the individual indicators identified would require Australian home care providers to undertake additional data collection. There were significant methodological limitations in the development of QI sets, particularly in the scientific evidence domain.

**Conclusions:**

This review identified important gaps in existing QI sets, which should be considered by policymakers, researchers, and other stakeholders when developing and applying QIs in the Australian setting.

**Policy Impact**

Australia is lagging behind other countries in implementing QIs in home‐ and community‐based aged care. This review identifies existing QI sets used internationally, identifies gaps in their development, and outlines considerations for relevant stakeholders, policymakers, and researchers in the future development and utilisation of QIs in the Australian context.

## 1 INTRODUCTION

Aged care services were provided to 1.3 million older Australians in 2019–20 at a cost to the government of $21.2 billion.^1^ As our population ages, the quality of aged care will play a critical role in determining the health and well‐being of an increasing number of older Australians and their informal carers. However, a series of inquiries, including the Royal Commission into Aged Care Quality and Safety, have highlighted a system suffering from significant funding and workforce pressures, and a stark absence of information on the quality of care provided.^2^

The vast majority of older Australians would prefer to age in place.^3^ Over three‐quarters of those using aged care services currently access support in their own homes and communities via the Home Care Packages Program (for people with complex needs who are eligible for residential aged care) and the Commonwealth Home Support Program (for entry‐level home support).^1^ While occupancy rates in residential aged care facilities have fallen over the last decade,^4^ the number of people using home care (HC) has tripled,^5^ and there is significant additional unmet demand for these services.^6^ Home‐ and community‐based aged care services provide vital support for basic health and care needs (such as meals, social support, and personal hygiene), support health service provision (e.g., transport to medical appointments), and can contribute to the prevention of falls, inappropriate hospital admissions, and premature entry to permanent residential aged care.^7^, ^8^, ^9^ The quality of the HC system thus has the potential to alleviate, or increase, pressures on other parts of the health and aged care systems.

Quality indicators (QIs) are standardised, evidence‐based measures of quality of care that are frequently utilised in health care settings to measure and track changes in quality, and to identify areas for improvement.^10^ According to the Donabedian model,^11^ information about the quality of care can be drawn from three categories: structure of care, processes, and outcomes. *Structure of care* refers to the characteristics of the setting where care is provided, such as the human resources or organisational structure; *process* refers to the activities carried out to provide care; and *outcomes* refers to the effects of care provided (both good and bad).^11^ A clear understanding of how these domains interact with and influence each other is important both in the development of quality indicators,^12^ and in supporting staff to understand the purpose of applying them. Quality indicators across these domains also need to be valid, reliable, feasible, acceptable, and sensitive for governments and providers to adequately monitor, compare, and drive improvements in care quality.^13^ Most importantly, quality indicator information needs to be publicly available if prospective care users are to make informed choices between providers.

Currently, in Australia, there has been no formal implementation of quality indicators for home‐ and community‐based aged care services. On 1 July 2019, the assessment and monitoring of care quality against a new single set of Aged Care Quality Standards by the Aged Care Quality and Safety Commission began.^14^ This standard consists of eight individual standards that all government‐subsidised aged care service providers must demonstrate for three‐yearly accreditation purposes. While these quality standards “set the rules” for the minimum quality of aged care, indicators enable that quality to be measured. Since 2019, residential aged care services have been required to report quarterly as part of the National Aged Care Mandatory Quality Indicator Program on three indicators: pressure injuries, use of physical restraint, and unplanned weight loss.^15^ Following criticisms of limited scope, two additional indicators (medication management, and falls and fractures) were added to the suite in July 2021.^15^ However, the Aged Care Royal Commission noted that a more comprehensive suite of indicators is needed across all care settings, and that this should include measurable outcomes of good person‐centred care such as quality of life.^2^ The development of indicators that are unique to home‐ and community‐based aged care is essential as the core goals (such as maintaining independence), setting (own home), funding model (individualised budget) and service delivery (intermittent visits) can differ markedly from residential aged care.

In 2017, the Australian Department of Health, in association with the (then) Aged Care Quality and Safety Agency, commissioned a pilot study of HC QIs.^16^ This included testing instruments with 740 people across three focus areas—goal attainment (using the Goal Attainment Scaling [GAS] tool), consumer experience (Your Experience of Services [YES] Survey), and quality of life (Adult Social Care Outcomes Tool SCT4 (ASCOT SCT4) and World Health Organisation Quality of Life questionnaire modified version for older people (WHOQOL‐BREF OLD)).^16^ However, the pilot HC QIs have not been taken up, and details on how these specific tools were selected and appraised in the pilot are not publicly available. Although the Aged Care Quality and Safety Commission is now piloting a HC consumer experience instrument via a Consumer Experience Interview,^17^ this does not allow for the assessment of either clinical or person‐centred outcomes. In late 2021, the Australian Department of Health commissioned another pilot of quality indicators for in‐home aged care to be undertaken in early 2022^18^—as of early 2022, information on the indicators considered or included has not yet been published. A rigorous quality indicator framework is needed for Australian HC, including both experienced and outcome measures.

Australia is lagging behind other countries in implementing QIs in HC. A recent systematic review by Joling and colleagues describes 17 quality indicator sets that have been developed for use with older people across different community care settings, including general practice and HC.^19^ However, a term frequently used to refer to personal care and support services in the home—social care—was not included in this review. Our paper aims to add to the literature by identifying and collating QIs that have been developed *and* are routinely used in HC settings internationally, and to critically appraise those indicator sets in terms of (i) the scientific quality of their development and (ii) coverage of key areas of care quality. By reviewing the current literature on the development and application of HC QIs in other countries, we aim to identify QI sets that may be feasible for implementation in Australia's home‐ and community‐based aged care.

## 2 METHODS

### 2.1 Data sources and searches

A systematic search of two electronic databases: MEDLINE (via Ovid) and Scopus, was performed in October 2020. The search strategy was developed in consultation with an experienced health research librarian and consisted of a combination of various search terms, including multiple terms for “quality indicators” and “home and community‐based aged care.” The search strategy is included in Appendix S1. These search terms were connected with appropriate Boolean connectors and involved the use of both keywords and Medical Subject Headings. A manual search of the reference list of eligible studies (snowballing technique) was also conducted to identify additional articles missed in the electronic databases mentioned above.

### 2.2 Eligibility criteria and study selection

Peer‐reviewed articles published from 1 January 2000 to 31 December 2019 were included. The decision to restrict the publication years to 2000 onwards was made to ensure that all QI sets were relevant and developed using more recent evidence. Articles were included if they were: (1) published in English, (2) focused on people aged 65 years and over, and (3) looked at the development of tools or instruments that had also been implemented to measure the quality of HC. Publications looking at residential aged care facilities/nursing homes were excluded. Publications that focused solely on people with cognitive impairment or those receiving palliation were also excluded as these settings require specific support and services, and the QI sets could not be applied across the entire sector. One researcher (HF) screened titles and abstracts of identified studies for eligibility, and two researchers (HF and MJ) independently assessed each article for inclusion at full‐text stage. Conflicts were discussed by researchers “HF” and “MJ” to reach a consensus, with a third researcher (JS) available to resolve any remaining conflicts.

### 2.3 Data extraction and categorisation

Two data extraction forms were developed to collate information about the HC QIs used by different countries. Relevant data from the included publications were extracted—author, year of publication, country, study aim, sample size, the total number of indicators in each QI set, and the development and validation process of the QI sets (Table 1).

**TABLE 1:** Summary of included home care QI sets

1st author, Year of publication	Country	Sample size	Name of QI set	Number of indicators	Development and validation process of the QI sets
Hirdes et al. (2004) [26]	Canada, United States	14, 293	interRAI’s first generation QI set	Total: 22 Structure: 0 Process: 3 Outcome: 19	Development of HC‐Qis (as a three‐nation effort) via extensive literature reviews, focus groups, and expert reviews Selection and review of HC‐QIs Empirical testing of approved HC‐QIs and development of risk adjustment based on cross‐national data
Morris et al. (2013) [20]	Canada, United States, Europe	335, 544	interRAI's second generation QI set	Total: 23 Structure: 0 Process: 1 Outcome: 22	Identification of candidate HC‐QIs, from interRAI's first‐generation QI set and additional sources Evaluation of candidate HC‐QIs in focus groups and one‐on‐one discussions by home care providers. HC‐QIs were then reviewed by interRAI's cross‐national program development committee Empirical testing of approved HC‐QIs and development of risk adjustment based on cross‐national data
Netten et al. (2012) [27]	England	1364	ASCOT (Adult Social Care Outcome Toolkit)	Total: 8 Structure: 0 Process: 0 Outcome: 8	Development of HC‐QIs based on previous work done on outcome measurement in social care via extensive literature review and consultation from service users Validity of questions tested via cognitive interviewing of service users, and validity testing of HC‐QIs Development and validity testing of utility weights

Abbreviations: HC, home care; QIs, quality indicators.

The individual QIs in the included publications were extracted and summarised in Table 2. Each QI was classified using the Donabedian model (structure, process, and outcome).^11^ This was done to identify potential gaps in the coverage of key areas of care quality in the QI sets. Each QI was also classified according to its content area (clinical/functional) and categorised as either prevalence or incidence indicators. Prevalence indicators were based on the proportion of individuals at follow‐up with a concern, whereas incidence indicators were based on the improvement or decline between a baseline and a follow‐up assessment.^20^

**TABLE 2:** Characteristics of home care QIs across the identified QI sets

Quality indicators	Donabedian model			Indicator type		Articles			Derivable from existing national datasets?^a^
	S	P	O	Prevalence^b^	Incidence^c^	Hirdes (2004) [26]	Morris (2013) [20]	Netten (2012) [27]	
Functional HC‐QIs									
ADLs			✓		✓	✓			NSAF
ADL improvement			✓	✓			✓		
ADL decline			✓	✓			✓		
IADL improvement			✓	✓			✓		
IADL decline			✓	✓			✓		
Rehab potential and no therapies		✓		✓		✓			MBS
Impaired locomotion in home			✓		✓	✓			NSAF
Difficulty in locomotion and no assistive device			✓	✓		✓			NSAF
Cognitive function			✓			✓			NSAF
Cognition improvement			✓		✓		✓		
Cognition decline			✓		✓		✓		
Difficulty in communication			✓			✓			NSAF
Communication improvement			✓		✓		✓		
Communication decline			✓				✓		
Clinical HC‐QIs									
Bladder incontinence			✓		✓	✓			NSAF
Bladder improvement			✓		✓		✓		
Bladder decline			✓		✓		✓		
Falls			✓	✓		✓	✓		NSAF, NHDC
Weight loss			✓	✓		✓	✓		NSAF, NHDC
Inadequate meals			✓	✓		✓			
Dehydration			✓	✓		✓			NHDC
No medication review by MD		✓		✓		✓			MBS
Delirium			✓	✓		✓			NSAF, NHDC
Injuries			✓	✓		✓	✓		NHDC
Skin ulcers			✓		✓	✓			NSAF, NHDC
Negative mood			✓	✓		✓			NSAF
Mood improvement			✓	✓			✓		
Mood decline			✓	✓			✓		
Pain improvement			✓		✓		✓		
Daily severe pain			✓	✓		✓	✓		NSAF
Pain not adequately controlled			✓	✓		✓	✓		NSAF
Social HC‐QIs									
Caregiver distress			✓				✓		NSAF
Alone and distressed			✓	✓		✓	✓		NSAF
Does not go out but used to			✓	✓			✓		
Social participation and involvement			✓	✓				✓	NSAF
Control over daily life			✓		✓			✓	
Personal cleanliness and comfort			✓	✓				✓	
Accommodation cleanliness and comfort			✓	✓				✓	
Food and drink			✓	✓				✓	
Safety			✓	✓				✓	NSAF
Neglect or abuse			✓	✓		✓			NSAF
Occupation			✓	✓				✓	
Dignity			✓	✓				✓	
Utilization HC‐QIs									
No flu vaccination		✓		✓		✓	✓		AIR
Hospital, emergency department, emergent care			✓	✓		✓	✓		NHDC

Abbreviations: ADLs, activities of daily living; AIR, Australian Immunisation Register; HC‐QIs, home care quality indicators; IADLs, instrumental activities of daily living; MBS, Medicare Benefits Schedule data collection; NHDC, National Hospitals Data Collection; NSAF, National Screening and Assessment Form; O, outcome; P, process; S, structure.

^a^ Indicates from which existing Australian national routinely collected dataset each QI could potentially be derived, as of mid‐2021. Blank = not derivable.

^b^ Prevalence, based on the proportion of individuals at follow‐up with a problem.

^c^ Incidence, based on the improvement or decline between a baseline and a follow‐up assessment.

Data dictionaries for existing national routinely collected datasets that could potentially be used to derive HC QIs in Australia were examined. This included data collected by Regional Assessment Services and Aged Care Assessment Teams using the National Screening and Assessment Form,^21^ the National Hospitals Data Collection,^22^ the Medicare Benefits Schedule data collection,^23^ and the Australian Immunisation Register.^24^ Each QI was classified according to whether it could be potentially “derivable” or not from these existing datasets without requiring additional data collection (i.e., whether information on a similar aspect of care is already collected elsewhere).

### 2.4 Methodological assessment

The Assessment of Indicators through Research and Evaluation (AIRE) instrument was used to critically appraise the QIs in eligible articles.^25^ The AIRE is a critical appraisal tool specifically for QIs that has been used extensively in the development and evaluation of QIs. It consists of four domains: (1) purpose, relevance, and organizational context, (2) stakeholder involvement, (3) scientific evidence, and (4) additional evidence, formulation, and usage. The four domains are further subdivided into 20 items which are listed in Table 3. The AIRE items are scored on a four‐point Likert scale; 1 = strongly disagree (confident that the criterion has not been fulfilled or no information was available), 2/3 = disagree/agree (unsure whether the criterion has been fulfilled), and 4 = strongly agree (confident that the criterion has been fulfilled). All eligible publications were appraised by two researchers (HF and MJ independently). The scores were then averaged between both researchers for each item and then summed and standardised to create the domain score, which ranges from 0% to 100%. A higher score (score above 50%) indicates the high methodological quality of the indicator set. Further details on the AIRE instrument and its scoring system can be found in the article by de Koning and colleagues.^25^

**TABLE 3:** Critical appraisal of home care QIs using the AIRE^25^ instrument

AIRE instrument items	Hirdes et al. [26]	Morris et al. [20]	Netten et al. [27]
Domain 1: Purpose, relevance, and organisational context	70%	47%	73%
1.1 The purpose of the indicator is described clearly	4	3	4
1.2 The criteria for selecting the topic of the indicator are described in detail	3	3	2
1.3 The organisational context of the indicator is described in detail	3.5	2	4
1.4 The quality domain the indicator addresses is described in detail	3	2	4
1.5 The health‐care process covered by the indicator is described and defined in detail	2	2	2
Domain 2: Stakeholder involvement	44%	61%	33%
2.1 The group developing the indicator includes individuals from relevant professional groups	3	4	2
2.2 Considering the purpose of the indicator, all relevant stakeholders have been involved at some stage of the development process	3	3.5	2
2.3 The indicator has been formally endorsed	1	1	2
Domain 3: Scientific evidence	6%	0%	22%
3.1 Systematic methods were used to search for scientific evidence	1	1	1
3.2 The indicator is based on recommendations from an evidence‐based guideline	1.5	1	2
3.3 The supporting evidence has been critically appraised	1	1	2
Domain 4: Additional evidence, formulation, and usage	67%	54%	41%
4.1 The numerator and denominator are described in detail	4	4	1
4.2 The target patient population of the indicator is defined clearly	3.5	2	2
4.3 A strategy for risk adjustment has been considered and described	4	4	1
4.4 The indicator measures what it is intended to measure (validity)	2	2	3
4.5 The indicator measures accurately and consistently (reliability)	1	1.5	3
4.6 The indicator has sufficient discriminative power	3.5	3	2
4.7 The indicator has been piloted in practice	1	1	2
4.8 The efforts needed for data collection have been considered	4	4	2
4.9 Specific instructions for presenting and interpreting the indicator results are provided	4	2	4

Abbreviations: AIRE, appraisal of indicators through research and evaluation.

## 3 RESULTS

### 3.1 Study selection

**FIGURE 1:**
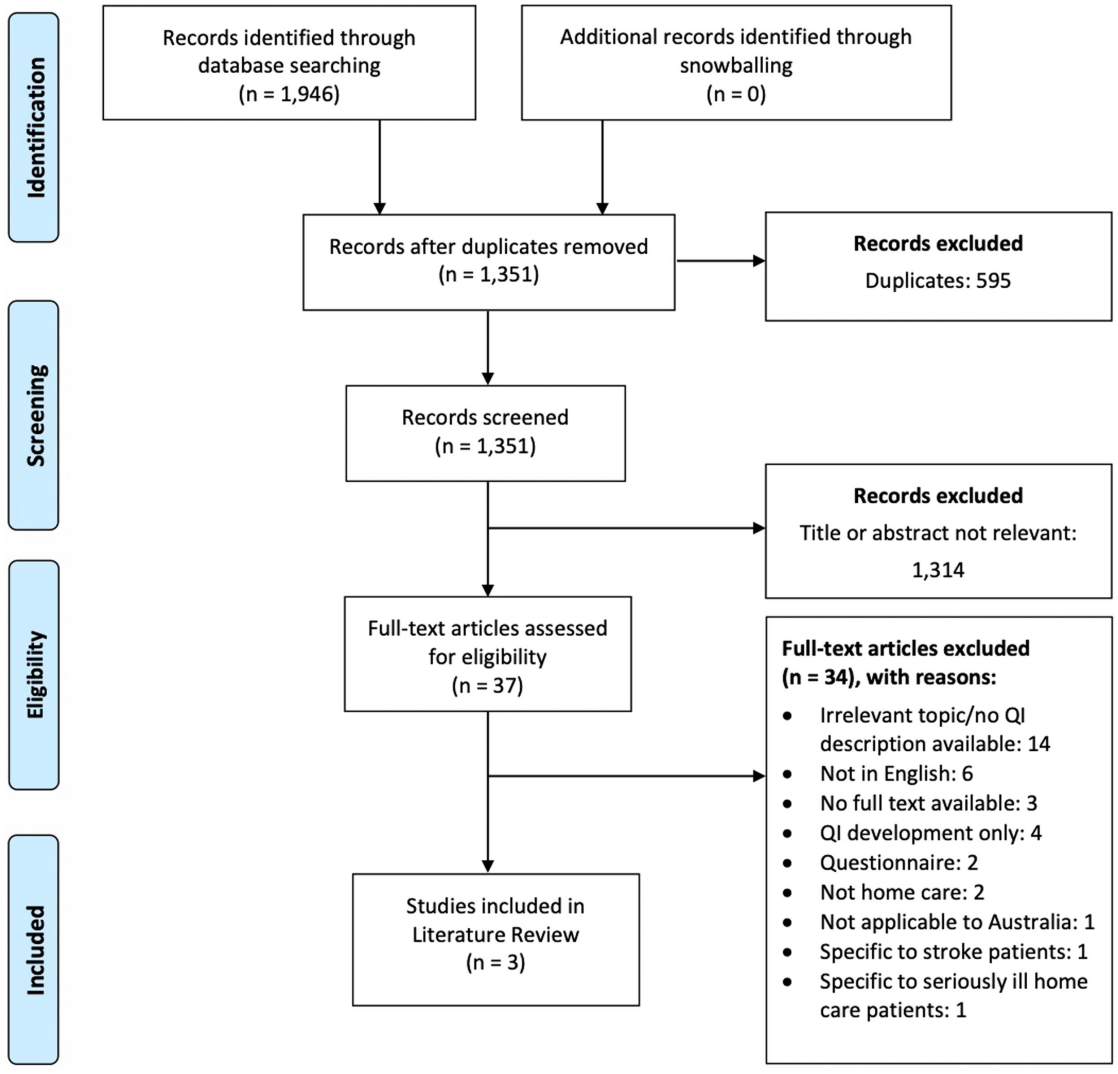
PRISMA flow diagram for study selection

The search identified a total of 1351 potentially relevant studies, across both databases (655 in MEDLINE, 1291 in Scopus, and 1351 after de‐duplicating). After screening the title and abstract of the identified studies, 37 papers were identified for full‐text review. Of the 37 papers, three papers with three unique sets of QIs met the selection criteria. Snowballing of relevant articles did not result in the identification of any additional papers for inclusion. The PRISMA flow diagram for the study selection process and reasons for exclusion are illustrated in Figure 1.

### 3.2 Study characteristics

The main characteristics of the papers included in this review are shown in Table 1. Of the three HC QI sets (HC‐QIs) identified, two were related QI sets; namely, the International Resident Assessment Instrument's (interRAI) first‐generation QI set^26^ and the interRAI's second‐generation QI set.^20^ The third QI set was the ASCOT,^27^ which looks at the development and validation of QIs focusing on the social care‐related quality of life. The first‐generation interRAI QI set was developed on a sample size of 14,293, with HC clients from Canada and the United States. The second‐generation interRAI QI set was developed on a large sample of 335,544 HC clients from Canada, the United States, and Europe. The ASCOT, however, was developed on a much smaller sample size of only 1364 clients from England. While the ASCOT has a self‐completion version that is widely used (ASCOT‐SCT4), the interRAI QI sets largely appear to involve assessments made by the provider. The three different QI sets cover a total of 45 unique quality measures. More information about the development and validation of each individual QI set is described in Appendix S2.

### 3.3 Characteristics of quality indicators

The quality indicators identified within each QI set are summarised in Table 2. The categorisation of each QI according to the Donabedian model and indicator type (prevalence vs. incidence) are also illustrated in Table 2. Of the 45 Qis, 42 were outcome measures (93%) and 3 were process measures (7%). None of the identified Qis measured the structure of care. The first‐generation interRAI QI set described by Hirdes et al. identified a total of 22 Qis, of which 19 were outcome measures and 3 measured processes of care.^26^ Morris et al.^20^ described the second‐generation interRAI QI set which included a total of 23 Qis, with only one QI measuring process of care. The Qis identified by the ASCOT were markedly different from the first‐ and second‐generation interRAI QI sets and consisted of 8 outcome measures, with no process or structure of care measures.^27^

The QIs fell into 4 broad categories: functional, clinical, social, and utilisation HC‐QIs. In total, there were 17 clinical, 14 functional, 12 social, and 2 utilisation HC‐QIs. There was considerable overlap in the QIs identified by both first‐ and second‐generation interRAI QI sets. The QIs shared between the two include falls, weight loss, injuries, daily severe pain, pain not adequately controlled, alone and distressed, no flu vaccination, and hospital, emergency department, and emergent care. The ASCOT focused on social care indicators only. Overall, the three QI sets contained 34 prevalence indicators (proportion of individuals at follow‐up), and 11 incidence indicators (change between baseline and follow‐up).

### 3.4 Derivability of QIs from existing Australian national routinely collected datasets

As of mid‐2021, 53% of the 45 QIs across the three sets could potentially be derived from existing national routinely collected datasets (see Table 2). A greater proportion of clinical and utilisation QIs had the potential to be derived (65% and 100%, respectively), compared to QIs in the functional (43%) and social categories (42%). Incidence (change) indicators would be more difficult to derive than prevalence indicators using existing national datasets.

### 3.5 Methodological quality of QI sets

The scores for methodological quality of each of the identified QI sets using the AIRE instrument are presented in Table 3. The methodological quality varied across the three QI sets. Both the first generation interRAI QI set and the ASCOT scored well in the first domain “Purpose, relevance and organisational context,” with the ASCOT scoring the highest (domain score of 73%). While the second‐generation interRAI QI set had lower scores for methodological quality in domain 1, it scored more highly in “Stakeholder involvement” (domain score of 61%) compared to the first generation interRAI QI set and the ASCOT, which both had low scores in that domain (44% and 33%, respectively). All three QI sets consistently scored poorly in the domain “Scientific evidence.” For “Additional evidence, formulation and usage,” both interRAI QI sets had higher methodological quality than the ASCOT, with a domain score of 67% for the first‐generation QI set and 54% for the second‐generation QI set. The ASCOT scored particularly poorly in domain items 4.1 and 4.3, as it did not describe the numerator and denominator of the indicators in detail and the strategy for risk adjustment was also not described in detail.

## 4 DISCUSSION

In this critical literature review, three sets of HC QIs developed and used internationally were identified—namely, interRAI's first‐generation QI set, interRAI's second‐generation QI set, and the ASCOT. Of the 45 unique QIs identified, the majority were outcome measures (93%), with only three measuring care processes (7%), and no indicators measuring quality in terms of the structure of care (for example, wait times to access care). There were significant limitations in some areas of methodological quality in the development of the QI sets, particularly in the domain of scientific evidence. Both interRAI QI sets focus on clinical and functional aspects of care, while the ASCOT has a greater emphasis on person‐centred measures of social needs and quality of life. Nearly half of the individual indicators identified would require Australian HC providers to undertake additional data collection. The limitations of existing QI sets should be considered by policymakers and strengthened during the testing and implementation phases of HC QIs in Australia.

One of the key gaps in the coverage of QIs identified by this review is quality measures for assessing the structure of care. Structure indicators encompass provider‐level issues such as the amount and adequacy of facilities and equipment and can provide guidelines on organisation structure such as staff qualifications or staffing ratios.^28^ They can also look at utilisation of system‐level resources, and characteristics that affect the systems' ability to meet the needs of individuals or communities,^29^ thus providing information on important aspects of quality such as accessible and equitable service provision. The identification of structural indicators is particularly important in the Australian context for monitoring the effectiveness of ongoing reforms to the HC system. Despite many calls to address the waiting list for HC packages, as of 30 June 2021, there remain nearly 80,000 older Australians waiting for government funding to access HC services at their approved level.^6^ Waiting time to access services, both at the government and individual provider level, was considered as an indicator in the 2017 Australian HC QI pilot, but ultimately was not included in the pilot because of high levels of missing data on waiting times.^16^ The Report on Government Services performance indicator framework identifies an additional structure of care measures such as the use of aged care by different groups, unmet need, and affordability.^1^ However, there are also significant gaps in available data for these measures.^1^ Australia currently provides data on two measures of long‐term care usage and resourcing for HC to the Organisation for Economic Co‐Operation and Development (OECD) for international comparison: the number of formal long‐term care workers at home and the number of long‐term care recipients at home.^30^ Further investing in the measurement of QIs that specifically assess the quality of HC at the structural level will be essential to ensure that resources increasingly put under pressure due to population ageing are equitably and effectively allocated.

The interRAI HC QIs are widely used internationally,^31^ and the second‐generation set is mandatory for home and community services in one of our closest neighbours, New Zealand. Data collected across six European countries using the interRAI suite have recently been used to develop a new benchmark methodology to identify best practices in HC (the “IBenC” project).^32^ However, the interRAI sets, while incorporating some social QIs, do not specifically assess experience and outcomes of care from the perspective of HC users. Although it is vital to measure clinical indicators of quality, older Australians report that person‐centred outcomes such as quality of life should be a central goal of aged care.^33^ The importance of quality of life measurement as part of comprehensive monitoring of aged care quality has been highlighted by the Aged Care Royal Commission,^2^ and is reflected in the inclusion of the ASCOT and WHOQOL‐BREF quality of life tool in the 2017 Australian HC quality indicator pilot.^16^ Previous research has also demonstrated that quality of life tools can be feasibly integrated into Australian aged care settings.^34^ However, restricting client views to the measurement of quality of life may fail to capture the full picture of service quality. The services' marketing literature often identifies two types of service quality: technical and functional quality.^35^ Technical quality refers to the delivery or outcome of the service (*what* is offered and received), while functional quality focuses on *how* the service is offered to and received by the customer (for example, experience of the service). Whilst the review mostly identified tools that focus on technical quality, there are other available person‐centred tools that focus on customer experience and should be considered in the comprehensive evaluation of HC quality. One of these identified during the process of undertaking this review was the Consumer Quality Index, developed over a decade ago in the Netherlands to measure client experiences with long‐term care.^36^ The Consumer Experience Interview pilot undertaken by the Aged Care Quality and Safety Commission further shows the increasing shift towards putting older people at the centre of their care.^17^ Incorporating consumer voices both in the QI data that is collected and in the wider implementation of HC QIs is central to ensuring that quality measurement improves the lives of those for whom the system is designed. These processes should be guided by evidence‐based approaches, including using appropriate methods for developing multidimensional and context‐specific service quality models that can capture the range of domains of service quality that are important to consumers.^37^

An area of QI development specifically addressed by the AIRE critical appraisal instrument is the effort needed for data collection. Utilising routinely collected data to measure QIs has significant appeal in reducing the data collection burden, particularly in settings such as Australian aged care where staffing resources are limited. The Registry of Senior Australians has recently developed an outcome monitoring system for residential aged care, consisting of 12 indicators to monitor care quality and safety utilising administrative health and aged care datasets.^38^ Electronic information systems that contain data generated during day‐to‐day care delivery are also becoming increasingly common in Australian aged care, which may allow for more readily accessible and up‐to‐date indicator derivation.^39^ These systems also allow for capturing important antecedent characteristics (individual and environmental risk factors), which should be considered in the development of valid quality measures and in allowing risk‐adjusted comparison between providers.^12^ However, the core goals of HC in maintaining independence and improving quality of life means it is unlikely a full set of measures could be developed from existing routinely collected data alone.

In their 2006 paper, the OECD expert health working group noted that indicator sets should be based on three main criteria: (i) the importance of what is being measured; (ii) the feasibility/cost of obtaining data; and (iii) the scientific soundness of the measure.^40^ Driving improvements in care delivery requires tight, evidence‐based links between outcomes and care processes.^41^ A key finding from this review is that the three identified QI sets are largely not based on strong evidence. Just over half of the indicators could be derived from existing Australian datasets. In addition, the minimal number of process measures across the QI sets means that providers with less skilled staff may not be supported to determine which specific aspects of the care are problematic and thus how to improve quality. Thus, more work is needed to ensure the validity, acceptability, reliability, and sensitivity of any of the QI sets, and their ability to change practice and improve outcomes for older Australians, before investing significant resources in their widespread application.

The Australian Community Care Outcomes Measure, which uses the ASCOT tool with additional demographic data, has been trialled in Australia with 200 clients, showing promising results in ease of use^42^ and potential for modified use with clients from culturally and linguistically diverse backgrounds.^43^ A recent study evaluating the implementation of the ICECAP‐O tool for over 1100 Australian HC clients found it could sensitively identify variations between service outlets in quality of life after adjusting for key client factors.^34^ A new tool, the Quality of Life‐Aged Care Consumers,^44^ has also recently been developed and tested specifically for the Australian population, based on qualitative interviews with community aged care users exploring the quality of life characteristics of importance to them.^45^ The rigorous piloting and evaluation processes for these tools, as well as other identified tools,^46^ can act as a guide for future field tests of QI sets in Australian HC.

### 4.1 Limitations

This review does not provide an overview of all possible QI sets that could be applied to Australian care. A number of QI sets that focus on specific populations, for example, those with dementia, as well as those developed in non‐English speaking countries were excluded from our review and might lead to a focus on developing QI sets that are tailored to specific systems. We did not include a number of QI sets that have been developed but never widely applied, such as the Home Care Quality Assessment Index developed in Japan.^47^ A grey literature search was also not completed for this review. Thus, QI sets that are used in practice but had no peer‐reviewed literature on their development, such as the Sweden's “Elderly Guide,”^48^ were also not included, to ensure QI sets had received appropriate scrutiny of their validity by experts. In addition, the methodological quality of the included QI sets may be underestimated due to the lack of detail provided in the publications. Nevertheless, this review has identified, critically appraised, and considered key issues in the future development and application of internationally relevant QI sets for Australian HC.

## 5 CONCLUSIONS

This critical literature review identified three QI sets developed and used to measure HC quality for older adults internationally. The implementation of an existing and widely used QI set in Australia such as interRAI would allow for international comparison but would need to be supplemented with additional indicators focusing on person‐centred experience and outcome measures of importance to older adults such as quality of life. In addition, there is a lack of strong evidence for the identified QI sets, and a gap in indicators measuring quality in terms of the structure of care. Thus, candidate QIs should be extensively tested with a population representative of Australian HC users to ensure acceptability, validity, and reliability before investing resources in their widespread implementation, particularly where QIs cannot be calculated from existing routinely collected data sources. All of these factors should be considered by relevant stakeholders, policymakers, and researchers in the development and utilisation of QIs for Australian HC.

## CONFLICTS OF INTEREST

The authors declare that Joyce Siette is an Associate Editor for the Australasian Journal on Ageing. All authors declare no other relationships, including financial or professional, which may pose a competing interest.

## Supporting information


Appendix S1
