# Supplementary material for: Quality indicators for home‐ and community‐based aged care: A critical literature review to inform policy directions
Source: Australas J Ageing. 2022 Jul 3;41(3):383–95. doi: 10.1111/ajag.13103 (PMC9542125; doi:10.1111/ajag.13103)
Supplement: Supplementary file 1 — Appendix S1 [file AJAG-41-383-s001.docx]

**Appendix S1**

***Search strategy:***

[“Quality indicators” OR “Quality improvement” OR “Quality assurance” OR “Health care quality, assess and evaluation” OR “Outcome and Process assessment” OR “Quality of health care” OR “Performance measure” OR “Outcome measure”] AND [“Home care” OR “Home care services” OR “Home-based aged care” OR “Community-based aged care” OR “In-home care” OR “Social care” OR “Home support” OR “Home health care”] AND [“Develop*”]

**Appendix S2**

***interRAI’s first generation quality indicator set***

The development and validation process of interRAI’s first generation QI set was described by Hirdes et. al.^26^ It was a three-nation effort, involving researchers, clinicians, and policy makers from Canada, United States and Japan. Data from 3,041 Canadian and 11,252 United States home care clients were used to evaluate the chosen HC-QIs. The development of HC-QIs was done by first identifying candidate QIs used in HC and in various other settings via an extensive literature review. Additional candidate QIs were identified by having expert meetings and focus groups with health professionals and older adults. This resulted in the identification of 73 candidate QIs. These HC-QIs were then ranked by five collaborating research centres based on the appropriateness of each QI. However, the ranking process and decision-making process were not discussed in detail in the article. Upon review, QIs with a relative frequency of less than 5% or more than 95% were excluded. The only exceptions to this were dehydration and abuse, due to their clinical importance. Of the 73 candidate HC-QIs, 22 were chosen to be further examined. The risk adjustment of the chosen QIs for interRAI’s first generation QI set is further examined by Dalby et. al.^49^

***interRAI’s second generation quality indicator set***

The development and validation process of interRAI’s second generation QI set was described by Morris et. al.^20^ Similarly to the development of interRAI’s first generation QI set, the first step is the identification of candidate QIs. This was achieved by including interRAI’s first generation QI set and QIs drawn from QI lists the interRAI considered for home care, post-acute care, and long-term care. This resulted in the identification of 64 candidate QIs. These QIs were then empirically tested based on a sample of 335,544 home care clients from Canada, United States, and Europe. Candidate QIs with a prevalence of less than 3% were excluded. In addition, QIs with poor clinical applicability were excluded (i.e., pressure ulcers, dehydration, delirium, and severe behavioural manifestations). Good quality measures are those with wide variation in scores, thus scores of the best performing sites were compared with scores of the worst performing sites; the comparison used scores at the 95^th^ and 5^th^ percentiles, to avoid comparing extreme outliers. Indicators with a less than two-fold difference in scores were excluded, as the variation is not wide enough. The remaining QIs were then evaluated. This involved working with the HC providers via focus groups and one-on-one discussions. Additionally, feedback regarding the earlier HC-QIs were provided by government-officials and HC providers. The HC-QIs were then reviewed by interRAI’s cross-national program development committee. Quality measures that were approved by less than 70% of the committee were excluded. The finalised HC-QIs were then risk-adjusted.

***Adult Social Care Outcomes Toolkit (ASCOT)***

The development and validation process of the ASCOT was described by Netten et. al.^27^ In contrast to interRAI’s first- and second-generation QI sets, the ASCOT looks at developing QIs that measure social care outcomes. The quality measures of the ASCOT were based on previous and parallel work done on the development of the ASCOT and the national Adult Social Care Survey. The final set of QIs were tested on a sample of 1,364 people from the 25 service user groups as a pilot postal survey.
